# Examining the need & potential for biomedical engineering to strengthen health care delivery for displaced populations & victims of conflict

**DOI:** 10.1186/s13031-017-0122-0

**Published:** 2017-11-01

**Authors:** Devika Nadkarni, Imad Elhajj, Zaher Dawy, Hala Ghattas, Muhammad H. Zaman

**Affiliations:** 1 Boston University College of Engineering, 44 Cummington Mall, Boston, 02215 USA; 2 American University of Beirut, Department of Electrical and Computer Engineering, Beirut, 1107 2020 Lebanon; 3 American University of Beirut, Epidemiology and Public Health Department, Beirut, 1107 2020 Lebanon; 4 Boston University College of Engineering, Department of Biomedical Engineering, 36 Cummington Mall, Boston, 02215 USA

**Keywords:** Biomedical engineering, Systems engineering, Displaced populations, Medical technology, Refugee health, Health systems

## Abstract

Conflict and the subsequent displacement of populations creates unique challenges in the delivery of quality health care to the affected population. Equitable access to quality care demands a multi-pronged strategy with a growing need, and role, for technological innovation to address these challenges. While there have been significant contributions towards alleviating the burden of conflict via data informatics and analytics, communication technology, and geographic information systems, little has been done within biomedical engineering. This article elaborates on the causes for gaps in biomedical innovation for refugee populations affected by conflict, tackles preconceived notions, takes stock of recent developments in promising technologies to address these challenges, and identifies tangible action items to create a stronger and sustainable pipeline for biomedical technological innovation to improve the health and well-being of an increasing group of vulnerable people around the world.

## Background

Refugees, internally displaced persons, and other victims of conflict are regarded to have among the poorest health outcomes in developing countries. These populations typically face a wide range of challenges in accessing adequate, quality medical care. The challenges presented in this paper do not occur in every situation, but are salient issues that these populations face in numerous regions and capture general trends. As far as possible, for each challenge we have specified the contexts in which they occur, so as not to overgeneralize. Multiple frameworks have been developed to describe the direct and indirect pathways that lead to adverse health outcomes for patients [1, 2]. Acute illnesses, chronic conditions, and the intergenerational effects on health due to conflict as well as the burden these have on health systems have been described in previous studies [1–3].

Here, we extend on this analysis by examining gaps for which health technology may be developed to address them [4]. There is extensive literature on the breakdown of health systems, the gaps and barriers to accessing care among mothers and children, the need for improved mental health services, and the continuous threat of infectious diseases [3, 5–17]. Our goal is not to review these challenges but to discuss why improved health technology is needed in humanitarian settings, and why it is so hard to develop and implement.

We consider health technologies to be a “broad category of interventions that reduce malnutrition, improve sanitation, and increase safety on roads” as well as “technologies specifically designed to prevent, diagnose, or treat illness, from the highly specific …to the more widely applicable” [4]. As such, in this work “humanitarian health technology” would focus on health technology developed for use within conflict, conflict-affected, and other humanitarian zones. We use the term “biomedical innovation” as the creation and development of health technology. The reasons for the gap in innovation towards humanitarian health technologies are numerous. Development of health technologies for conflict and conflict-affected regions possesses unique obstacles – including limited access to field assessments, ethical considerations, and rapidly changing environments – that deters pursuit of further innovation [18]. There has been tremendous progress in the development of digital technology for these contexts, however gaps remain where needs of the health system are outside the scope of software and communication tools. Biomedical engineering may fill these gaps through health systems level innovation.

This paper seeks to discuss what role biomedical engineering can play for the future of humanitarian health and the opportunities for its future application. We elaborate on examples of technologies that have been developed for use in conflict and conflict-affected regions that improve delivery of health care either through direct patient use, distribution of medical resources, or improved training of medical personnel. The technologies described here illustrate broadly the current state of humanitarian health technology, and are not intended to be an exhaustive list. While the focus of this paper is on solutions for forced migration due to conflict and social unrest, we recognize that technologies developed for forced migration in response to natural disasters have shown promise and relevance for conflict situations [19, 20].

## Analysis of the gaps and opportunities for the future

We now turn our discussion to the causes for gaps in technology development and focus on both the barriers and the opportunities for innovations. Despite the growing refugee population, and a global increase in conflict and subsequent forced migration, technological solutions that address short and long-term health challenges suffer from serious gaps. The pipeline of new innovations is relatively weak, and few solutions make it to the point of implementation. There are several major reasons for these limitations on the translation of innovation and technology. The first one is lack of awareness among developers, innovators and technology experts. While there have been recent meetings and conferences on humanitarian innovation, these are often few and far between, and do not translate into broad awareness beyond those who are already engaged in the research [21–23]. Research institutions and institutions of higher education offer few, if any, courses that create awareness among scholars, researchers and engineering students to address the glaring gaps in point-of-care solutions in conflict situations. Engagement of technology developers with on-the-ground health and humanitarian workers is minimal; therefore, the needs of the field are often not translated into technologies developed in the labs. Interdisciplinary efforts that bring technology developers in regular contact with the front-line workers remain weak and limited.

Coupled with the lack of awareness is the issue of lack of financial incentives, both for individuals and innovation enterprises [24]. These range from lack of direct investment in niche technologies for conflict situations to unavailability of grants for research and development. The issue of funding is not just acute for early development but also implementation and field-optimization that require engagement of multiple stakeholders, testing on-site and optimization based on user experience and feedback [24]. In the absence of awareness and funding and with the lack of convenient access to impacted populations, the ability of researchers to come up with optimized solutions is minimal.

Finally, the absence of trained staff in conflict areas and refugee settlements, to maintain and appropriately use equipment, adds a further layer of complexity and challenge [25, 26]. Lack of training of the front-line workers, on one hand, means that they are unable to fully engage technology developers and on the other hand, increases the chances of technology malfunction and disrepair. While there have been some efforts in the recent past to increase technical competency of the front-line workers in using digital and point-of-care technology, a lot more needs to be done to increase their awareness, engagement and ability to fully benefit from technological innovations.

## The path ahead in creating solutions

With an increase in global conflicts and an ever increasing number of refugees and those who are forced from their homes, there is a critical need to develop and implement innovative solutions to meet the health demands of those affected. These solutions need to be cognizant of socio-economic realities, respectful of traditions and culture and implementable in complex and difficult settings. While technology may not solve all of these challenges, it has an important role to play. Recent past has seen a surge of digital and information technology solutions being developed and implemented, yet solutions that are rooted in biomedical engineering and address point-of-care needs in diagnosis, management and treatment, have been relatively few. As we look towards the future, we offer four concrete approaches to increase development and implementation of these point-of-care and point-of-need solutions.

### Awareness and success stories

While each conflict environment and refugee settlement is unique, and requires solutions optimized for that scenario, there have been successes in other complex environments via effective point-of-care solutions. These include natural disasters such as floods and earthquakes, and more broadly for global health settings. Solutions such as rapid diagnostics, incubators that are rugged and robust and prosthetics that meet the needs of the affected population have made substantial impact [4]. Key lessons from those situations include awareness among the engineering community about the needs, complexity of the challenges, fundraising opportunities, multi-sectoral engagement and partnership between those who develop to those who work at the frontlines. Similar strategies are needed to address gaps in technology development for conflict and refugee settlements. A significant number of refugees now live in urban areas, outside the refugee settlements. An understanding of their needs in accessing health services would require a more sophisticated understanding of the local challenges. This would once again, require a stronger partnership between various sectors working in refugee health and rehabilitation and an engagement that is rooted on both the ground realities and the ability to create solutions that are specific and sensitive.

### Increasing frontline innovation

Creating solutions at the point-of-care is now becoming a possibility through advancements in manufacturing, 3D printing and paper based diagnostics. Additionally, combined with digital technologies for better imaging, microscopy and analysis, it is now within the realm of possibility to create solutions that are sensitive and specific, and also independent of long, complicated and expensive supply chains. Investments in innovations that can be created closer to the point-of-care and point-of-need can decrease cost, increase sustainability, provide economic support to local communities and improve the local adoption.

### Early stage research incentives

The incentives for engineering, technology and science faculty, students and researchers to focus on developing novel solutions for conflict situations and refugee crises remain weak. Even with limited funding, a focus is often on the applied and the implementation, with little incentives for development, early stage investigation and optimization. The lack of research support significantly affects the ability of scholars to devote their efforts in coming up with new and novel solutions that may have the ability to make a sustainable impact. Grants that are focused, yet cognizant of the research pipeline will, on one hand, increase awareness, and on the other, bring new researchers to the fore to develop novel and high impact solutions.

### A multi-sectoral approach for funding, development and sustainability

Given the recent political shift towards nativism, there are strong reasons to expect a decline in international aid. Therefore, new models of investment have to be developed. In this regard, a multi-sectoral approach, that combines public and private funding, NGOs and foundations, individual philanthropists and large multi-national corporations, is needed in order to discover, develop and deliver solutions. Once again, there are positive examples from innovations in global health that have developed mechanisms that range from grants to direct investment from multiple sectors to create and implement solutions. These include multi-stake holder grant mechanisms such as Saving Lives at Birth, Grand Challenges mechanisms in multiple countries, the Grand Challenge on Ebola, the development of Global Health Investment Fund to name a few [27–30]. The Global Humanitarian Lab also offers opportunities for funding and partnership that supports and accelerates bottom-up innovation [31]. A similar strategy that is both multi-sectoral and diverse in its origin and impact can bridge some of the current gaps in point-of-care and point of need solutions.

## Conclusions

Conflict and displacement due to conflict result in adverse outcomes across many different areas of health – including but not limited to maternal and child health, communicable diseases, and mental health. Technology towards improved delivery of medical care and training, distribution of medical supplies, and manufacturing of medical equipment have been developed – however, substantial gaps in reducing the burden of illness and increasing access to medical care still exist. We believe that while this gap presents an enormous challenge in coming up with new solutions for conflict environments, there is also a significant opportunity to develop and design solutions that are sustainable and can lead to discovery that may impact regions beyond those that are currently affected through reverse innovation and sharing of design paradigms [32, 33]. A concise, multi-scale effort that starts with individuals in the classroom or the research lab, and extends to those who are most vulnerable due to conflict or forced migration, can deliver on that promise.

## Abbreviations

UNFPA: United Nations Population Fund
UNHCR: United Nations High Commission for Refugees

## References

[1] Tatah L, Delbiso TD, Rodriguez-Llanes JM, Gil Cuesta J, Guha-Sapir D, Orach C, et al. Impact of refugees on local health systems: a difference-in-differences analysis in Cameroon. PLoS One. 2016; 10.1371/journal.pone.0168820. 10.1371/journal.pone.0168820 PMC5161383 27992563

[2] Devakumar D, Birch M, Osrin D, Sondorp E, Wells JCK. The intergenerational effects of war on the health of children. BMC Med. 2014; 10.1186/1741-7015-12-57. 10.1186/1741-7015-12-57 PMC3997818 24694212

[3] IOM (Institute of Medicine). The Causes and Impacts of Neglected Tropical and Zoonotic Diseases : Opportunities for Integrated Intervention Strategies. Natl. Acad. Press. 2011. 21977543

[4] Howitt P, Darzi A, Yang GZ, Ashrafian H, Atun R, Barlow J (2012). Technologies for global health. Lancet.

[5] Footer KHA, Rubenstein LSA (2013). Human rights approach to health care in conflict. Int. Rev Red Cross.

[6] Physicians for Human Rights. Syria: Attacks on Doctors, Patients, and Hospitals. 2011. https://s3.amazonaws.com/PHR_Reports/syria-attacks-on-drs-patients-hospitals-final-2011.pdf. Accessed 21 Sept 2017.

[7] Coghlan B, Brennan R, Ngoy P, Dofara D, Otto B, Clements M (2006). Mortality in the Democratic Republic of Congo: a nationwide survey. Lancet.

[8] The Brookings Institution. Protecting Internally Displaced Persons: A Manual for Law and Policy Makers. 2008. http://www.unhcr.org/en-us/protection/idps/50f955599/protecting-internal-displaced-persons-manual-law-policy-makers-october.html. Accssed 21 September 2017.

[9] Al Gasseer N, Dresden E, Keeney G, Warren N (2004). Status of women and infants in complex humanitarian emergencies. J Midwifery Womens Health.

[10] Black BO, Bouanchaud PA, Bignall JK, Simpson E, Gupta M (2014). Reproductive health during conflict. Obstet Gynaecol.

[11] O’Hare BAM, Southall DP (2007). First do no harm: the impact of recent armed conflict on maternal and child health in sub-Saharan Africa. J R Soc Med.

[12] Prasad AN, Prasad PL (2009). Children in conflict zones. Med J Armed Forces India.

[13] Food Security Information Network (2017). Global report on food crises 2017.

[14] Moss WJ, Ramakrishnan M, Storms D, Henderson Siegle A, Weiss WM, Lejnev I (2006). Child health in complex emergencies. Bull world heal. Organ.

[15] Machel G (1996). Promotion and protection of the rights of children: impact of armed conflict on children.

[16] Murthy RS, Lakshminarayana R (2006). Mental health consequences of war: a brief review of research findings. World Psychiatry.

[17] Gele AA, Bjune GA (2010). Armed conflicts have an impact on the spread of tuberculosis: the case of the Somali regional state of Ethiopia. Confl Heal.

[18] Brophy-Williams S, Hardman J, Leaning J, Mieier P, Olaffson G, Pham P, et al. World Disasters Report: Focus on technology and the future of humanitarian action. Lyon; 2013. http://www.ifrc.org/en/publications-and-reports/world-disasters-report/world-disasters-report-2013. Accessed 21 September 2017.

[19] Fernandes A, Zaman M. The role of biomedical engineering in disaster management in resource-limited settings. Bull World Health Organ. 2012;90 10.2471/BLT.12.104901 PMC3417780 22893748

[20] Brock TK, Mecozzi DM, Sumner S, Kost GJ (2011). Evidence-based point-of-care tests and device designs for disaster preparedness. Am J Disaster Med.

[21] Humanitarian Innovation Project. Humanitarian Innovation Conference | OXIP [Internet]. 2017. http://www.oxhip.org/events/hip2015. Accessed 21 Sept 2017.

[22] IEEE. IEEE Global Humanitarian Technology Conference [Internet]. 2017. Available from: http://2017.ieeeghtc.org/. Accessed 21 Sept 2017.

[23] Humanitarian Technology - Science, Systems, and Global Impact [Internet]. 2017. http://www.humanitariantechnology.org. Accessed 21 Sept 2017.

[24] El-Noush H, Silver K, Pamba A, Singer P. Innovating for women’s, children’s, and adolescents’ health. BMJ. 2015:49–52. 10.1136/bmj.h4151 26371217

[25] Richards-Kortum R. Biomedical Engineering for Global Health. Cambridge University Press; 2010.

[26] Perry L, Malkin R (2011). Effectiveness of medical equipment donations to improve health systems: how much medical equipment is broken in the developing world?. Med Biol Eng Comput.

[27] Saving Lives at Birth: A Grand Challenge for Development [Internet]. 2016. https://savinglivesatbirth.net. Accessed 21 Sept 2017.

[28] Grand Challenges. 2016. https://grandchallenges.org/#/map. Accessed 21 Sept 2017.

[29] Ebola F (2015). A grand challenge for development.

[30] Global Health Investment Advisors LLC. Global Health Investment Fund [Internet]. 2016. http://www.ghif.com. Accessed 21 Sept 2017.

[31] Global Humanitarian Lab. Global Humanitarian Lab - Accelerating Human Innovation. 2017. https://globalhumanitarianlab.org. Accessed 21 Sept 2017.

[32] Snowdon AW, Bassi H, Scarffe AD, Smith AD, Begin M, Eggertson L (2015). Reverse innovation: an opportunity for strengthening health systems. Glob Health.

[33] DePasse JW, Lee PT, Syed S, Dadwal V, Rutter P, Storr J (2013). A model for “reverse innovation” in health care. Global. Health.

